# Exploring the Remediation of Behavioral Disturbances of Spatial Cognition in Community-Dwelling Senior Citizens with Mild Cognitive Impairment via Innovative Technological Apparatus (BDSC-MCI Project): Protocol for a Prospective, Multi-Center Observational Study

**DOI:** 10.3390/jpm14020192

**Published:** 2024-02-08

**Authors:** Davide Maria Cammisuli, Cosimo Tuena, Giuseppe Riva, Claudia Repetto, Nikolai Axmacher, Varnan Chandreswaran, Valeria Isella, Simone Pomati, Stefano Zago, Teresa Difonzo, Giada Pavanello, Lorenzo Augusto Prete, Marco Stramba-Badiale, Alessandro Mauro, Stefania Cattaldo, Gianluca Castelnuovo

**Affiliations:** 1 Department of Psychology, Catholic University, 20123 Milan, Italy; davide.cammisuli1@unicatt.it (D.M.C.); claudia.repetto@unicatt.it (C.R.); 2 Applied Technology for Neuro-Psychology Lab, IRCCS Istituto Auxologico Italiano, 20145 Milan, Italy; cosimo.tuena@unicatt.it (C.T.); giuseppe.riva@unicatt.it (G.R.); 3 Human Technology Lab, Catholic University, 20145 Milan, Italy; 4 Department of Neuropsychology, Faculty of Psychology, Institute of Cognitive Neuroscience, Ruhr University, 44801 Bochum, Germanyvarnan.chandreswaran@ruhr-uni-bochum.de (V.C.); 5 Department of Neurology, School of Medicine, University of Milano-Bicocca, 20126 Milan, Italy; valeria.isella@unimib.it; 6 Milan Center for Neurosciences, 20133 Milan, Italy; 7 Neurology Unit, Luigi Sacco University Hospital, 20157 Milan, Italy; simone.pomati@asst-fbf-sacco.it; 8 Fondazione IRCCS Ca’ Granda, Ospedale Maggiore Policlinico, University of Milan, 20122 Milan, Italy; stefano.zago@unimi.it (S.Z.); teresa.difonzo@policlinico.mi.it (T.D.); 9 School of Specialization in Clinical Psychology, Catholic University, 20123 Milan, Italy; giada.pavanello01@icatt.it (G.P.); lorenzoaugusto.prete@unicatt.it (L.A.P.); 10 Department of Geriatrics and Cardiovascular Medicine, IRCCS Istituto Auxologico Italiano, 20145 Milan, Italy; stramba_badiale@auxologico.it; 11 “Rita Levi Montalcini” Department of Neurosciences, University of Turin, 10126 Turin, Italy; alessandro.mauro@unito.it; 12 Neurology and Neurorehabilitation Unit, IRCCS Istituto Auxologico Italiano, “San Giuseppe” Hospital, 33081 Piancavallo, Italy; 13 Clinic Neurobiology Laboratory, IRCCS Istituto Auxologico Italiano, “San Giuseppe” Hospital, 33081 Piancavallo, Italy; s.cattaldo@auxologico.it; 14 Clinical Psychology Research Laboratory, IRCCS Istituto Auxologico Italiano, 20149 Milan, Italy

**Keywords:** spatial navigation, Alzheimer’s disease, mild cognitive impairment, subjective cognitive decline, elderly, APOE, assistive technology

## Abstract

Spatial navigation (SN) has been reported to be one of the first cognitive domains to be affected in Alzheimer’s disease (AD), which occurs as a result of progressive neuropathology involving specific brain areas. Moreover, the epsilon 4 isoform of apolipoprotein-E (APOE-ε4) has been associated with both sporadic and familial late-onset AD, and patients with mild cognitive impairment (MCI) due to AD are more likely to progressively deteriorate. Spatial navigation performance will be examined on a sample of 76 community-dwelling senior citizens (25 healthy controls; 25 individuals with subjective cognitive decline (SCD); and 26 patients with MCI due to AD) via a virtual computer-based task (i.e., the AppleGame) and a naturalistic task (i.e., the Detour Navigation Test—modified version) for which a wearable device with sensors will be used for recording gait data and revealing physiological parameters that may be associated with spatial disorientation. We expect that patients with MCI due to AD and APOE-ε4 carriers will show altered SN performances compared to individuals with SCD and healthy controls in the experimental tasks, and that VR testing may predict ecological performance. Impaired SN performances in people at increased risk of developing AD may inform future cognitive rehabilitation protocols for counteracting spatial disorientation that may occur during elders’ traveling to unfamiliar locations. The research protocol has been approved by the Ethics Committee of the Istituto Auxologico Italiano. Findings will be published in peer-reviewed medical journals and discussed in national and international congresses.

## 1. Introduction

### 1.1. Spatial Navigation Processes

**Figure 1:**
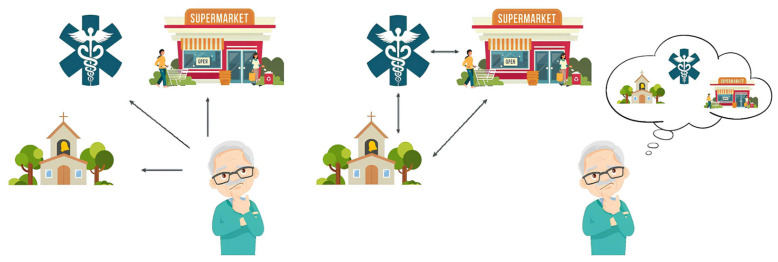
On the left, a representation of egocentric navigation based on the navigator; on the right, a representation of allocentric navigation based on the relationships among landmarks (designed by Freepik).

Navigation abilities include the precision at which spatial information is encoded from sensory experiences, the ability to build up spatial representations of external environments, and the efficacy by which they are used to guide navigational behavior. These abilities are highly interdependent, and typical navigational tasks bridge multiple levels [1]. Navigation in space is a complex ability including multiple cognitive and perceptual processes that are essential for the autonomy of individuals in their lives [2]. Specifically, spatial navigation (SN) can be defined as the ability of a human being to use multiple cues to plan and travel a route [3]. Cues that are fundamental for SN can be basically divided as follows: (i) self-motion ones, usually referred to idiothetic cues; and (ii) environmental ones, also known as allothetic cues. The former includes motor, vestibular information, and proprioceptive information used to track one’s self position and orientation; the latter comprises environmental (visual predominantly) information pertaining to stable space cues, such as landmarks and boundaries, that are used to estimate one’s position and orientation within the environment [4]. Crucially, both idiothetic and allothetic cues can inform and support spatial frames of reference. The first frame of reference is the egocentric representation, which is a body-dependent representation of the space; the second frame of reference is the allocentric representation, which is a body-independent (world-centered) representation of the environment [5] (Figure 1).

**Figure 2:**
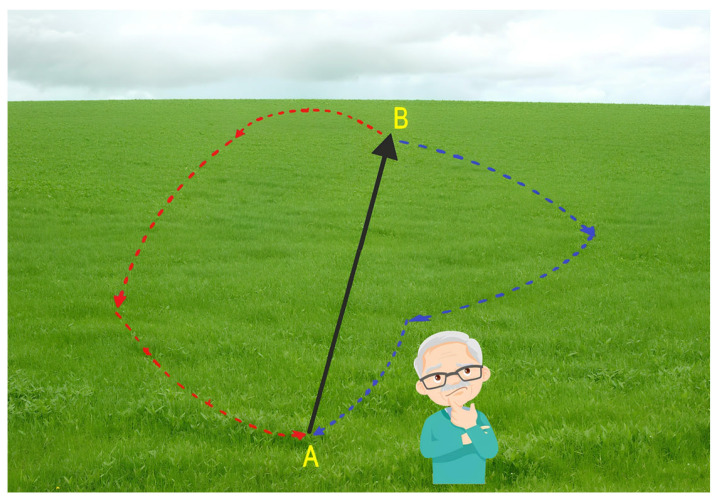
An example of a PPI virtual task: The navigator has to reach a destination point from a start position (black route). When he/she arrives, he/she is asked to come back to the start point without using any external support, potentially travelling many different routes, for example, red/blue routes (designed by Freepik).

The human navigation system naturally encourages the strategic translation of egocentric and allocentric information and the use of path integration. People ordinarily use egocentric navigation strategies during visual–spatial tasks such as walking a familiar route many times [6]. The egocentric navigation strategy is self-centered and involves the encoding of spatial information in relation to the position of the navigator. It is also based on different cues, allowing individuals to navigate as follows: perpetual processing of visual landmarks by bodily movements (e.g., “Turn left at the church”, “Turn right at the supermarket”, and then “Go straight to the pharmacy”), an endogenous integration of sensorimotor stimulation, vestibular information, and self-motion [6]. By contrast, when people travel a novel route, egocentric spatial representations are not available, and world-related navigational strategies are employed (i.e., allocentric navigation). They allow individuals to mentally represent a “cognitive map” of the environment based on the relationships among landmarks. This navigation strategy is also referred to as “wayfinding” [7]. Remarkably, they permit the travelling of alternative routes to come back to the starting point [6]. Further, when people are able to use urban environmental landmarks, they can successfully navigate thanks to the translation of spatial information from allocentric to egocentric strategies and vice versa (e.g., “I am 20 m from the pharmacy” and “The pharmacy is to the right of the supermarket”) (cf., [6]). Path integration, which is based on idiothetic cues, is the ability to transform internal information into a sense of location and orientation [8]. Self-motion cues (vestibular, proprioceptive, and motor information) are combined together to allow individuals to continuously estimate their position and orientation in space [9]. When participants walk blindfolded and with ear plugs, or when the environmental information is removed (with the exception of the optic flow alone), for example, in virtual reality tasks, it is possible to isolate idiothetic information from allothetic information [10]. Allothetic navigation, in turn, uses landmarks and boundaries to successfully navigate the environment [11], and pure allothetic navigation can be detected through desktop-based virtual reality tasks only [12]. Pure path integration (PPI) is particularly important because it permits the estimation of distances and the navigation of environments without using boundaries and landmarks. PPI can be studied both in virtual tasks, providing the navigator with a scenario bare of boundaries and landmarks (Figure 2), and in real-world settings thanks to the use of idiothetic indexes, as in the case of the triangle completion task [10]. However, in our daily experience, path integration and allothetic strategies are used jointly to derive location and direction. Some studies have shown that providing allothetic information during path integration tasks or vice versa can improve people’s orientation ([8,13,14]).

Lastly, a crucial aspect during SN is represented by spatial memory, which enables individuals to encode, store, and retrieve spatial information. Pure forms of memory for spatial information like a path are recognition, free recall, directions, and symbolic representations (e.g., map drawing). Conversely, wayfinding/shortcuts and path integration tasks over complex trajectories require a balanced used of purely navigation abilities (e.g., self-motion) and spatial memory [15]. Spatial tasks used to assess spatial memory performance predominantly rely on egocentric or allocentric representation. The former may include landmark recognition and sorting or asking participants to travel a previously learned route. The latter may include map-drawing and shortcut tasks [16,17].

### 1.2. Neural Correlates of Spatial Navigation

**Figure 3:**
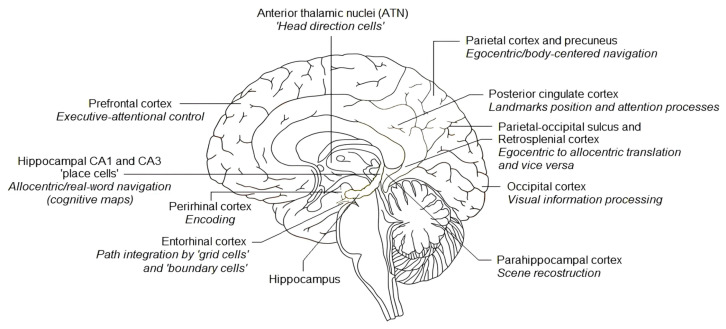
An anatomical illustration of brain areas supporting spatial navigation (designed by Freepik).

Advances in the investigation of neural correlates of human SN have shown that a large network of cognitive mechanisms, supported by different brain areas, is involved (Figure 3). The cerebral areas sub-serving egocentric and allocentric strategies interact with specific brain cells supporting SN, i.e., head direction (HD) cells, grid cells, boundary cells, and place cells (PC). The anterodorsal thalamic nucleus—included in Papez’s circuit—contains a large proportion of head direction (HD) cells [18] informing the individual as to in which direction he/she is heading. They maximally fire when the head of the person is facing in a specific direction with respect to the surrounding environment [18]. Grid cells are neurons of the entorhinal cortex that exhibit firing fields as triangular patterns tiling the environment [19]. Boundary cells, discovered in the medial entorhinal cortex, too, fire in response to fixed boundaries in the surrounding space, contributing to analyzing the limits of the environment [20]. The hippocampal formation in both rats and humans is involved in spatial navigation [21], and spatial memories are thought to be encoded and retrieved in the hippocampus through the activity of place cells [22]. Place cells fire when an animal visits specific regions of the environment, or “place fields” [23]. Specifically, hippocampal CA1 and CA3 place cells in the medial temporal lobe provide allocentric mental representations [24]. According to Puthusseryppady [25], novel environments are first encoded as egocentric representations by the parietal cortex. As one person continues to move within the environment, the HD cells and the grid cells provide information for brain path integration. The egocentric representations are then transformed into the allocentric representations by the retrosplenial cortex and the parietal-occipital sulcus, and this is combined with information provided by the boundary cells in order to generate and store “cognitive maps” in the hippocampus via the place cells. However, in other cognitive domains, such as executive functions, attention and working memory may play a critical role in SN performance [26].

### 1.3. Spatial Navigation in Alzheimer’s Disease

AD is a multifactorial neurodegenerative disease, resulting from complex interactions between genetic and environmental factors, that remains the leading cause of dementia to date. The proportion of people aged 65 years and over is growing rapidly worldwide, and it is estimated that by 2060, this age group will account nearly for 24% to 29% of the population in Western Europe and the United States [27], representing a health emergency due to social and sanitary costs. AD is characterized by progressive deterioration of cognitive functions, with episodic memory loss and spatial disorientation as the main hallmarks. No causal therapies for AD are currently available, although drug treatment efforts are progressing [28]. Thus, advances in biomarkers and behavioral tests for identifying individuals at increased risk of developing AD represent a crucial target of current research and therapeutics. Spatial navigation, or the ability to determine and maintain a trajectory from one place to another, gradually declines in the course of physiological aging [29,30,31], and spatial disorientation (SD) represents a core feature of pathological aging, especially AD [32]. Although cognitive decline precedes overt AD, pathological changes may even begin years before there are any overt behavioral impairments, and new treatments are being sought to modify this process [33]. Indeed, it has been proposed that subjective cognitive decline (SCD) (i.e., a self-experienced decline without objective cognitive impairment) might appear at the end of the preclinical phase of AD [34]. SN complaints are frequent symptoms not only in AD but also in SCD and MCI, and can be detected by specifically designed questionnaires [35] or by virtual and real-world navigation tasks [36]. SCD is gaining increased prominence in neurodegenerative research as a potential marker for future mild cognitive impairment (MCI) due to AD [37], a condition that represents the prodromal state of dementia associated to the disease. Approximately 90% of MCI patients with pathologic cerebrospinal fluid biomarker levels at baseline develop AD within 9 to 10 years [38]. Deficits in spatial orientation appear early in the course of the AD continuum [16]. AD is mainly characterized by the presence of beta-amyloid protein plaques and neurofibrillary tangles of hyperphosphorylated tau protein. According to Laczó et al. [7], amyloid-β accumulation first emerges in the neocortical regions and, early on, affects the retrosplenial cortex, while tau pathology first emerges in the transentorhinal cortex (Braak stage I) and spreads to the posteromedial entorhinal cortex and the hippocampus (Braak stage II and III) and finally to the posterior cortical regions (Braak stage IV). Neurodegeneration and brain atrophy parallel the pattern of tau pathology spreading [39]. Degeneration of the entorhinal cortex as a critical brain district supporting SN represents an initial stage of typical AD [40], and a profound loss of its neurons occurs in very mild AD [41]. Neurofibrillary AD pathology in the entorhinal cortex and hippocampus also represent the neuropathological substrate of memory decline [42] and defective PPI [43]. Finally, tau abnormalities in the entorhinal cortex can already be observed in adults under the age of thirty [44], especially in those carrying the Apolipoprotein-E(APOE)-ε4 allele [45].

Finally, it is worth mentioning the extent to which the visual cortex modulates SN functioning. In fact, navigation is strongly driven by vision, and visuospatial deficits of cortical origin may play a critical role in AD [46]. In this neurological condition, there are a variety of visual symptoms, including visual field coverage, contrast sensitivity, color discrimination, visuospatial perception, and visual processing speed [47]. This may impair the cortical representation of visual space. Thus, as visual symptoms can occur early in AD, it is possible that the evaluation of visual cortex changes may aid in the early detection of neurodegeneration [47]. Accordingly, emerging evidence pertaining to AD neuropathology has indicated that neurofibrillary tangles and neuritic senile plaques steadily increase from the primary to associative visual cortex [47].

### 1.4. Genetic Risk Factors for Alzheimer’s Disease: Role of the Apolipoprotein-E [APOE]

Beyond increasing age, the ε4 allele of the apolipoprotein-E gene represents the most important risk factor for AD, providing an opportunity for assessing subclinical alterations of behavior at very early disease stages. APOE is centrally involved in lipoprotein metabolism and other biological functions [48]. ApoE-lipoproteins are involved in lipids transport into bloodstream, and they also bind to hydrophobic amyloid-β (Aβ) peptide, which is thought to initiate toxic events that lead to synaptic dysfunction and neurodegeneration in AD [49]. APOE genotype is determined by three main alleles, ε2, ε3 and ε4, resulting in six possible combinations (i.e., ε2/2, ε2/3, ε2/4, ε3/3, ε3/4, and ε4/4). Population frequency of the ε4 allele is approximately 14%, the ε2 allele 6%, and the ε3 allele 78% [50]. The presence of the ε4 allele is associated with an increased risk of cognitive decline during normal aging [50]. The APOE-ε4 allele is also an established risk factor for neurodegenerative conditions, such as AD [51,52] as opposed to the ε2 allele that offers protection against the disease [53,54]. Individuals carrying one APOE-ε4 allele are at threefold increased risk and those carrying two ApoE-ε4 alleles are at more than tenfold increased risk of developing AD [47]. Remarkably, cognitively normal subjects who are homozygous for the ε4 allele have reduced glucose metabolism in the same regions of the brain as in patients with probable AD [55].

In a previous investigation using a VR test (i.e., The AppleGame), we provided evidence of a specific deficit of PPI decades before potential disease onset in AD risk carriers, specifically when the recruitment of compensatory navigational strategies via supportive spatial cues from posterior cingulate/retrosplenial cortex was disabled [43].

### 1.5. Cognitive Markers Associated to AD Neuropathology

Episodic memory loss—as the most sensitive and specific cognitive marker—has long been considered “the gold standard” for the diagnosis of cognitive impairment due to AD, although its manifestation in healthy aging is quite common [56]. Early detection of incipient dementia pathophysiology is difficult during aging [6], and neuropsychologists have to schedule periodic follow-up assessments at clinics to confirm the progression of cognitive impairment and restrictions in personal and instrumental autonomy to consider a patient to have fulfilled the diagnostic criteria of dementia. Evidence from the literature indicates that SN deficits can indicate individuals at higher risk of developing AD early [29], with strong implications for timely intervention. SN deficits have the potential for early detection of cognitive alterations in topographical orientation since they have higher specificity in distinguishing AD from healthy aging [57,58], as well as from other dementia types [59,60,61]. Further, animal models of AD pathophysiology have reported on how navigation-specific brain areas are primarily affected, rather than episodic memory ones [62,63]. SN is one of the earliest cognitive domains to be impaired in patients with AD that may result in SD. It refers to instances where AD patients are unaware of where they are and are unable to reach their intended location [64]. It behaviorally manifests in AD patients making navigation errors when they go out of their community, which can lead to an increased risk of going missing in both familiar and unfamiliar environments [65], with negative consequences for personal safety, including potential injuries and, in the worst cases, even death [66]. Remarkably, incidents of the patient going missing restrict patient’s autonomy and self-efficacy and increase the caregiver’s burden [67]. In line with previous studies [64,68], AD patients may present with “moments of hesitation” while walking in their familiar environments. A “moment of hesitation” is defined as the participant slowing down/stopping and looking around to aid orientation or verbally admitting that he/she is unsure about their whereabouts. This is consistent with the fact that when participants exhibit hesitant walking, more variation would be seen in their step intervals, as compared to when they are more confident in wayfinding (cf., [25]). Further, when an elderly person loses their sense of direction, he/she moves to come back as soon as possible to reach the original start point and may present with some distressing symptoms and spatial anxiety [69], potentially influencing cardiac and respiratory physiological functions. Considering SN dysfunction as a cognitive biomarker of AD, interventions that may help in preventing SD using the most advanced wearable technology may contribute to detecting and treating the initial manifestation of the disease. SD has been specifically described in AD and MCI patients as deficits in route learning, free recall or recall of the temporal order of the landmarks, landmarks recognition and location on a 2D map, route drawing, and evaluating directions [70]. However, to date, no study in the literature has pointed out physiological correlates (e.g., physiological stress indexes) that might be associated with them. Deterioration in SN, manifested in poor hippocampus-dependent allocentric navigation, may also occur before the clinical onset of dementia [71]. A study investigating navigation skills as a function of the APOE allele found that patients with amnestic MCI and APOE-ε4-positive allele performed more poorly on both allocentric and egocentric tests than amnestic MCI patients with the APOE-ε4-negative allele [72]. Further, it has been demonstrated that virtual and ecological SN tasks can differentiate the SN performances of amyloid-positive MCI patients and amyloid-negative MCI patients ([16,73]). Finally, from the earliest stages of AD, the presence of specific impairment in the ability to encode and store an allocentric hippocampal representation and then to translate it into an egocentric parietal representation has been recently observed, and it has been proposed as a specific cognitive process that may be useful in supporting the recall of spatial scenarios too [74], which could be impaired even in premorbid AD.

### 1.6. Technological Solutions to Avoid Spatial Disorientation for Cognitively Impaired Older Adults

New technological solutions, including digital devices, sensors, and VR, represent promising means for assessment, monitoring, and intervention in AD population [75], but scientific investigations in this research field are still lacking. Advances in technology currently provide the potential for designing innovative solutions for evaluating, monitoring, and treating AD-related symptoms, both cognitive and non-cognitive ones [75]. Co-designing cutting-edge technological solutions by using brainstorming, focus groups, questionnaires, detailed interviews, and surveys with all the relevant stakeholders, including physicians, formal and informal caregivers, staff members, and bioengineers, makes the evaluation of the impact of one technological application more effective and ensures that the system meets the quality and sustainability standards for people with AD and those who take care of them [75]. By following a nudge theoretical approach [76], thanks to the collaboration of the Catholic University of the Sacred Heart, Bicocca University, and IRCCS Auxologico Institute in Milan (Italy), we set up the SENIOR (SystEm of Nudge theory based ICT applications for OldeR citizens) project, an advanced technology coaching system able to collect and integrate physiological, psychological, and behavioral data, with the final aim of interacting with community-dwelling senior citizens with MCI and providing them with personalized feedback for maintaining independence from caregivers and improving their lifestyles [77]. Cognitively impaired older adults’ mobility may be reduced due to frailty, sensory impairments, functional limitations, and comorbidities. Digital navigation devices can overcome these deficits, providing a sense of situation-awareness and offering proactive navigational assistance using remote monitoring by physicians and caregivers. A wearable monitoring solution designed for older adults in a continuous, non-invasive way is thus necessary in order to detect SD physiological correlates via the use of digital sensors. According to Ghosh et al. [78], wearables-based sensing of everyday behavior can be used to provide ecologically valid digital biomarkers of AD. In their study, the authors used GPS tracking to measure ecological outdoor behavior in people with AD and showed that they significantly differed from controls in specific mobility domains.

### 1.7. Aim of the Study Protocol

The first endpoint of the research is to explore differences in SN performances among healthy older adults, individuals with SCD, and patients with MCI due to AD on a VR task (i.e., the “AppleGame”) and on a naturalistic task (i.e., the Detour Navigation Test-modified version, DNT-mv) using an innovative technological apparatus recording gait data and revealing physiological parameters that may be associated with SD.

The second endpoint of the research is to verify if examinees’ performances in such a well-characterized laboratory-based SN-testing task may predict impairment in a real-world context.

## 2. Materials and Methods

### 2.1. Design, Study Population, Recruiting Centers and Trial Registration

This is a multi-center prospective observational study. A sample of outpatients with SCD and MCI due to AD will be enrolled from three sites in Lombardy (Italian Region): “San Gerardo” Hospital (Monza), “Luigi Sacco” University Hospital (Milan), and Fondazione IRCSS Ca’ Granda Ospedale Maggiore Policlinico (Milan, Italy).

Individuals with SCD will be detected according to Jessen and colleagues’ criteria [79] while prodromal AD will be defined according to Dubois and colleagues’ criteria [80], respectively. Healthy older adults will be enrolled through advertisements distributed in social centers for senior citizens in Milan (Italy) and through popular science events (conferences, webinars) concerning research in AD prevention. The three sites currently use the neuropsychological batteries reported in the Appendix A. Healthy older adults will be assessed by the experimenters at the Department of Psychology, Catholic University (Milan, Italy), by adopting the same neuropsychological battery as the IRCSS Ca’ Granda Ospedale Maggiore Policlinico (Milan, Italy). Family medical history of AD was collected during clinical interview along with laboratory tests results and neuroimaging findings for outpatients with SCD and MCI due toAD.

For the whole sample, inclusion criteria will involve the following: age from 65 to 85 years; education not less than 5 years; basic ICT skills. All participants will be excluded from the study according to the presence of visual, hearing, or motor impairment significantly interfering with SN tasks; neurological/psychiatric disease or other medical conditions preventing SN; history of alcohol or drugs addiction; intake of psychotropic drugs; and presence of dementia. Recruitment will end on 29 February 2024.

With the aim of obtaining results that can be generalized, sociodemographic and clinical features of the sample to be recruited will be analyzed among the research team members prior to the sample enrollment according to epidemiological studies carried out on the Italian elderly population, specifically including MCI patients [81,82,83] and individuals with SCD [84].

The Uniform Resource Locator for the clinical trial registration is the following one: https://classic.clinicaltrials.gov/ct2/show/NCT05944601 (identifier: NCT05944601) (accessed on 13 July 2023).

### 2.2. Genetic and Psychological Evaluation

All participants will be genotyped for the APOE polymorphisms by collecting genetic material extracted from buccal brushes using the Gentra Puregene^®^ (40723, Hilden, Germany) specific assay. The collected samples will be analyzed in the Neuropathology and Clinical Neurobiology Laboratory of “San Giuseppe Hospital” in Piancavallo (Verbania, Italy), IRCCS Istituto Auxologico Italiano. All participants will be blinded towards APOE genotype.

Subjective strategies of SN and personal satisfaction for wearable technology will be evaluated via the Subjective Spatial Navigation Complaints (SSNC) questionnaire [35] and the Tele-healthcare Satisfaction Questionnaire (TSQ-WT) [85], respectively, which have previously undergone a back-translation process for Italian culture adaptation.

### 2.3. Experimental Navigation Tasks

#### 2.3.1. Virtual Navigation Test

In a comfortable and silent room under the experimenters’ supervision, all participants of the research will first perform the short version (i.e., 56 trials) of a computer-based SN task, i.e., the AppleGame [43], specifically designed by the researchers for older adults (≥65 years) as a measure of virtual PI. This VR task comprises 8 practice trials followed by 16 trials for each of the three subtasks. They differ with regard to the presence or absence of supportive spatial cues: the PPI subtask without any supportive cue; the boundary PI (BPI) subtask with a circular boundary; and the landmark PI (LPI) subtask with an intra-maze landmark (e.g., a lighthouse) close to the center of the virtual circular arena (please see Figure 1 in [43] for a complete representation of the experimental paradigm). For each trial, the participants have to collect a basket (i.e., start phase) and try to remember its location (i.e., the goal location). After a navigation phase toward a variable number of trees (1 to 5, i.e., “the outgoing phase”) which disappear after having been reached, the participants have to find their way back to the goal location (i.e., “the incoming phase”) after they have collected an apple under the last tree. The “outgoing distance” and the “incoming distance” will be recorded. Path integration performance for each subtask will be assessed as the distance between the correct goal location and the response location, i.e., the drop error, constituting the score of the “AppleGame”.

#### 2.3.2. Naturalistic Navigation Test

**Figure 4:**
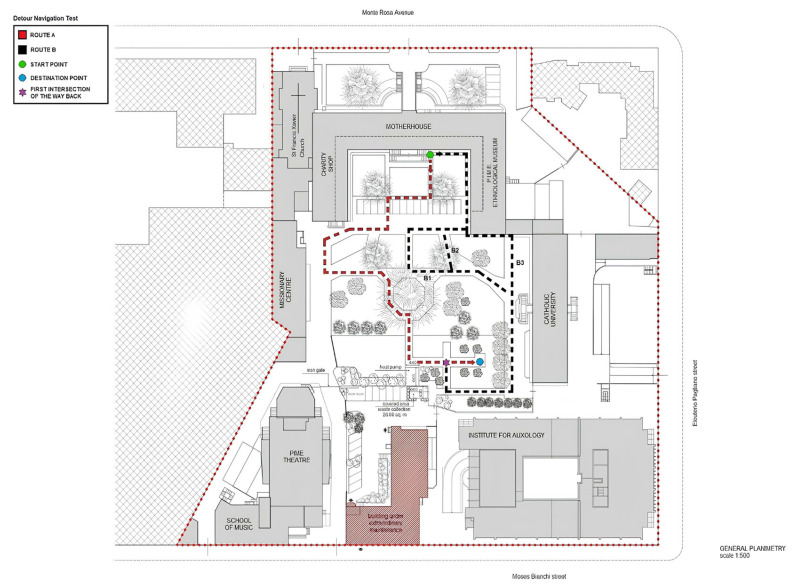
General planimetry of the Pontificio Istituto Missioni Estere (PIME, 81 Monte Rosa Street, Milano, Italy) with the experimental ecological task: the Detour Navigation Test—modified version (DNT-mv) on the outdoor urban garden. B1= first option for Route B retour; B2= second option for Route B retour; B3= third option for Route B retour.

All participants will then undergo a modified version of the “Detour Navigation Test” (DNT-mv) [64] in a naturalistic environment, i.e., an urban park. In contrast to the original one, the version we created takes into account the fact that our participants do not present with dementia; they retain some ability to navigate in urban unfamiliar environments in comparison to AD patients, for which the exploration of places outside home is very critical [25]. The DNT-mv will be performed in the outdoor garden of the Pontificio Istituto Missioni Estere—PIME (81, Monterosa Street, Milan, Italy), a 1000 m^2^ arena (20 × 50 m^2^) (Figure 4), in order to guarantee that MCI patients, SCD individuals, and healthy elderly controls have a safe environment to explore. The experimenter and his staff consisting of clinical psychologists will follow the elderly participants for their safety, providing instructions for the tasks to be performed without giving any feedback on the participants’ correctness.

**Figure 5:**
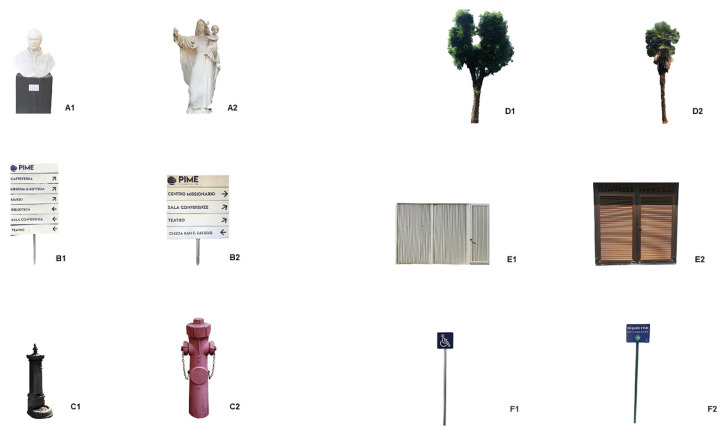
PIME landmarks: hits in the left column; distractors in the right column. (**A1**–**F1**) correct landmarks; (**A2**–**F2**) distractors similar to the landmarks.

The participants are asked to complete a path from a start point (Figure 4, green point) to a destination point (Figure 4, blue point) in *Route A* (80.5 m) (Figure 4, dotted red line, on a scale of 1 to 500) in the PIME outdoor garden under the experimenter’s guidance. An interfering cognitive task mimicking distracting urban stimuli (e.g., people chattering, cars horns, background noise, pedestrian crossing, recognizing someone on the street, receiving a phone call, etc.) will be provided by a smartwatch worn by the participants, who are required to touch the screen when it displays a specific colored shape among different shapes presented with an interval of 15 s from each other. During this path, they are also required to remember all the existing landmarks as a cognitive exercise useful for the evaluation of spatial episodic memory. Once at the destination, they are asked to put in order the encountered landmarks (Figure 5), randomly presented as a written naming list on the experimenter’s smartphone App. In case of a sudden technology breakdown, an algebraic calculation (i.e., “*Please, subtract 7 from 100 and keep going until I stop you*”) and a different landmarks reordering (i.e., presentation with flash cards) will be provided by the experimenters as replacements. The landmarks will be mixed up with distractors in order to detect hits and false alarms. Then, they are required to navigate back to the original start point using the same route. This first task requires participants to predominantly utilize an egocentric strategy based on a navigator-centered representation of the environment.

Later, the participants will be required to reach the destination point of the encoded *Route A* again. However, at the first intersection of the way back (Figure 4, purple asterisk), unknown to them, they will be asked to find an alternative route to come back (*Route B^1^*, 94 m; *Route B^2^*, 78.5 m; *Route B^3^*, 74 m) that does not overlap with *Route A* at all (Figure 4, dotted black lines). This second task requires participants to predominantly utilize an allocentric strategy based on a formed cognitive map of the garden.

**Figure 6:**
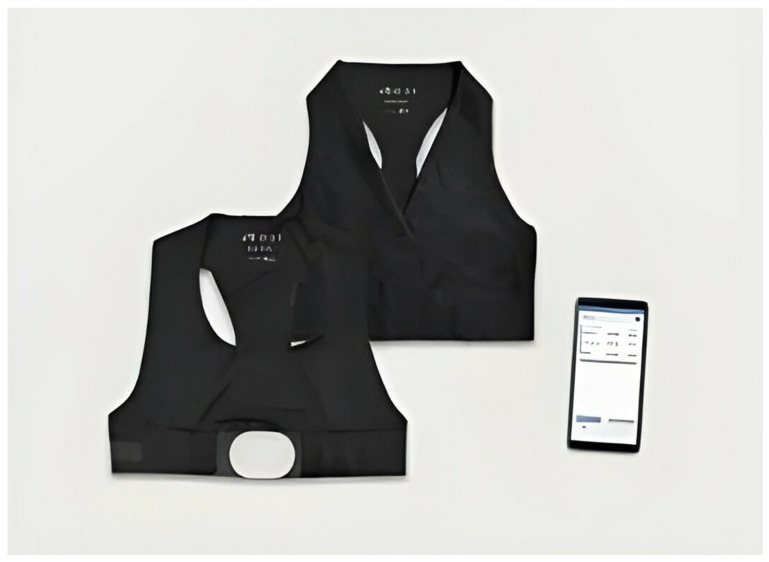
Howdy Senior system components. The electronic unit (white) detects heart rate, respiratory rate, and body position. It is equipped with five automatic snaps on the back and communicates with the Howdy app via Bluetooth. The electronic unit can be combined with different textile units: male/female top with sensors and belt. The electronic unit and textile unit are connected using the automatic snaps. The Howdy Apps works on smartphones and tablets running Android 8.0 or later.

Before the navigation of the urban garden, all participants will equip the Howdy Senior^©^ (Comftech S.r.l., Monza, Italy) device (Figure 6), a wearable monitoring system. All participants will be recorded according to cardiac and respiratory parameters *at rest* for five minutes and during the naturalistic experiment (Table 1). These data will be available thanks to the instant reports produced by the connected “Howdy Senior App” set up on the experimenter’s smartphone.

### 2.4. Statistical Analysis

Data distribution will be first checked on collected variables by assessing skewness and kurtosis values, by performing normality and heteroscedasticity tests, and by visually inspecting histogram and Q–Q plots. Based on data meeting or non-linear model assumption, either parametrical or non-parametrical analyses will be performed. By means of the R marco *pwr* [86], the minimal sample size has been set as 76 participant, based on a power analysis for a multiple regression model that took into account the following parameters: α = 0.05; 1-β = 0.9; *f*^2^ = 0.15 (medium effect size, in order to be conservative) [87]; and numbers of predictors: 3 (age, APOE genotypes, and belonging to each group, with the last two predictors as dummy variables).

We will detect the route disorientation scores, i.e., WTs and MsH, as already described in Puthysserypaddy et al. [64]. Accordingly, all the violations in terms of deviations from the pre-established return path (i.e., movement at an intersection onto a different path as compared to the original one, or non-roadable walkways) will be written down by the experimenter as WTs in a pre-set paper grid for *Route A*. The overlaps with the *Route A* return path, and, likewise, non-roadable walkways will be written down by the experimenter as WTs in a pre-set paper grid for Route *B*. Further, we will detect the MsH as the participant either slows down/stops and looks around to aid orientation or verbally admits to the experimenter that he/she is unsure about his/her whereabouts, both for *Route A* and the *Route B* return path.

In addition to the experimenter visually identifying and recording the frequency of the MsH with a stopwatch and a pre-set paper grid, we will measure this variable more objectively using the Howdy Senior integrated accelerometer. For this purpose, we will use the motion sensor of the sensorial body measuring the individual’s linear acceleration in the three axes (i.e., x, y, z). To segment the magnitude signals, according to Schaat et al. [88], we will adopt time intervals of 10 s, with an overlap of 5 s between subsequent intervals because a 10 s segment is sufficiently long to capture multiple steps during walking, thus revealing important behavioral changes. In this way, we will detect the MsH as a signal flattened along the three axes. Further, given that physiological parameters may alter depending on the MsH, we will also investigate the relationship between step variability and co-occurring time-dependent cardiac and respiratory parameters detected by the Howdy App, expressed as mean values, via Pearson’s correlation coefficients or their non-parametric equivalent (i.e., Spearman’s rank order correlation). Moreover, we will run a one-way ANOVA (or its non-parametric equivalent) to compare performances of the three subgroups on gait parameters collected through the Aptive app and the SSNC questionnaire. In order to explore potential association between specific neurocognitive functions and SN impaired abilities, Pearson correlations or their non-parametric equivalent (i.e., Spearman’s rank order correlation) will be further performed between the AppleGame/DNT-mv results and the neuropsychological tests used in the sending centers, especially visual–constructive skills and executive functions (see Appendix A). Given the difference of the neuropsychological battery used in the sending centers, we will group tests according to the primary cognitive domain involved and we will divide the scores obtained in a dichotomous way for each participant according to the reported cut-off values in the test manuals, i.e., 0 = pathological performance; 1 = normal performance.

Finally, a linear/generalized linear regression analysis will be run, taking into account the drop errors of the AppleGame subtasks—considered one at a time—as independent variables, and the total number of “wrong turns” (WTs)/total number of “moments of hesitation” (MsH) (in seconds) of the DNT-mv as dependent variables.

## 3. Discussion

In relation to the first endpoint, we expect that patients with MCI due to AD will show significantly more SN deficits than healthy controls and individuals with SCD, both in the AppleGame and in the DNT-mv, in terms of higher drop errors on BPI, LPI, and PPI and higher amounts of WTs and MsH, respectively. Virtual and ecological deficits may reflect a cognitive inefficiency of the entorhinal cortex, where AD neuropathology manifests early [25]. It has also been demonstrated that virtual and ecological SN tasks can detect the SN alteration of amyloid-positive MCI patients [89,90]. According to Colmant et al. [91], we also presume that APOE-ε4 carriers will report a PPI deficit in the VR task differently from non-carriers, regardless of subgroup.

Furthermore, we also anticipate that significant physiological parameters modifications (particularly, respiratory function) may be particularly associated with MsH, given that anxiety rather than depression significantly influences SN abilities in non-demented elders [92]. Starting from difficulties in spatial orientation evaluated in naturalistic experiments able to modify gait [93], we finally assume that patients with MCI due to AD will perform worse than SCD and healthy controls in terms of “Time” and “Speed”, and report significant differences in “Direction”, all variables collected by the Howdy Aptive app in the open-space naturalistic task.

In relation to the second endpoint of the research, we assume that the drop errors obtained in the VR task may predict the route disorientation scores of the naturalistic task. In particular, we expect a causal relationship between the BPI drop error of the AppleGame and the *Route A* return path of the DNT-mv, as well as between the LPI drop error of the AppleGame and the *Route B* return path of the DNT-mv, because they mainly rely on egocentric and on allocentric navigation, respectively. A recent systematic review and meta-analysis [44,94] has outlined that the association of navigational tasks performed in virtual and real environments needs to be deeply clarified. In light of this, we believe that the AppleGame could represent a validated technique with which to detect, in laboratory, early SN deficits in populations at risk of dementia conversion to provide high-quality, time-limited diagnosis. It is a cost-effective tool able to predict real-life SN impairment that should be routinely used in clinical practice.

However, our research protocol presents some limitations. Urban park conditions (e.g., presence of potential distractors) might influence the performance of the study participants in the naturalistic task, and the technology used may breaks down in relation to the GPS signal power. Moreover, the frequency of APOE-ε4 carriers, especially in patients with MCI due to AD, may be unsatisfactory due to specific demographical variables of our European country [95]. Although the APOE-ε4 represents the strongest genetic risk factors for AD [54], as genetic research has proceeded, studies have found suggestive links between late-onset AD and a number of other genes, such as ABCA7, CLU, CR1, PICALM, PDL3, TREMZ, and SORL1 [96,97], which might be relevant in influencing SN performances, too.

Nevertheless, we first estimate the impact of the present research project in terms of an innovative technological SN assessment in senior citizens at high risk of developing AD dementia, with relevant implications for maintaining independent spatial orientation in daily life activities. In fact, this kind of smart technological apparatus (i.e., a body T-shirt with sensorial devices and a smartphone with digital health Apps) is able to alert caregivers when their loved ones with cognitive decline are required to navigate unfamiliar locations alone, and it constitutes a potential means of remotely monitoring them.

## 4. Conclusions

Spatial navigation is a complex cognitive ability that is essential for the independence, quality of life, and safety of the elderly. Spatial navigation has been reported to be one of the first cognitive domains to be affected in AD, which occurs as a result of progressive neuropathology involving specific brain areas. Thanks to the use of a comprehensive assessment (i.e., virtual and naturalistic evaluation) implemented by an advanced technological apparatus, our research protocol constitutes an innovative method of investigating SN performances in participants at increased risk of developing AD, potentially revealing the employment of compensatory strategies of navigational guidance able to resist neuropathological damage, and may suggest the definition of specific cognitive remediation techniques in order to improve visuospatial skills. Findings will be disseminated through scientific articles as well as attendance at national and international congresses or academic lectures/seminars/workshops.

## Figures and Tables

**Table 1:** Cardiac, respiratory, and accelerometer parameters collected by the Howdy Senior App.

**Cardiac system functions** *Time domain over RR intervals parameters*	avgRR: mean of R2R interval; SDRR: standard deviation of R2R interval; avgHR: mean of Heart rate; SDHR: standard deviation of Heart rate; MSSD: mean of square successive differences between normal heartbeat; RMSSD: root mean square of square of successive differences between normal heartbeat; lnRMSSD: natural log of RMSSD; hrvScore: normalized lnRMSSD value using as scaling factor the value 6.5; RR50: number of successive absolute difference greater than 50 ms; pRR50: percentage of number of successive absolute difference greater than 50 ms over the number of elements in the analyzed window; SD1: standard deviation along the minor axes of the Poincare plot; SD2: standard deviation along the major axes of the Poincarè plot; Stress index.
**Cardiac system functions** *Frequencies domain over RR intervals parameters*	LF: power in the low frequency band affected by sympathetic nervous system (SNS); HF: power in the high frequency band affected by parasympathetic nervous system (PNS); LF/HF: ratio between the power in low and high frequency band.
**Respiratory system functions**	avgBR: mean of BR; SDBR: standard deviation of BR.
**Accelerometer parameters**	AccXavg, AccYavg, AccZavg: mean of accelerations for X, Y, Z axes; AccXsd, AccYsd AccZsd: standard deviation of accelerations for X, Y, Z axes; AccXrms, AccYrms, AccZrms: root mean square of accelerations for X, Y, Z axes.

The Howdy Senior system has been recently implemented by the “Aptive” App capable of recording GPS data during the naturalistic experiment. These data include “Time” as the number of minutes to complete the route; “Latitude”, “Longitude”, “Altitude”, and “Speed” as estimated by the body integrated accelerometer for calculating steps variability; and “Direction” corresponding to gait orientation with values ranging from 0° to 360°, where 0° corresponds to the north, 90° to the east, 180° to the south, and 270° to the west. Thanks to the Aptive App, a GPX tracking of participants’ body movements around the urban environment will be registered, too.

## Data Availability

The datasets presented in this article are not readily available because they are part of an ongoing study.

## Appendix A

**Table A1:** Neuropsychological tests used in the recruiting centers.

	“San Gerardo” Hospital Monza, Italy	“Luigi Sacco” Hospital Milan, Italy	Policlinico Hospital, Milan, Italy
**Global cognition**	Mini Mental State Examination (Magni et al., 1996) [98]	Mini Mental State Examination (Foderaro et al., 2022) [99] or Montreal Cognitive Assessment (Aiello et al., 2022) [100]	Mini Mental State Examination (Magni et al., 1996) [98]
**Memory** ***Short-term memory auditory–verbal***	Digit span backward (Monaco et al., 2013) [101] Digit span forward (Monaco et al., 2013) [101]		Digit Span Backward (Monaco et al., 2013) [101] Digit Span Forward (Monaco et al., 2013) [101]
***Visuospatial***			Corsi Span Backward (Monaco et al., 2013) [101] Corsi Span Forward (2013) [101]
***Long-term memory auditory–verbal***	List of semantically related words (Mauri et al., 1997) [102]	Free and Cued Selective Reminding Test (Girtler et al., 2015) [103]	Pairs Associate Learning (Normative data of the Hospital) Memory of prose (Normative data of the Hospital)
***Visuospatial***	Rey–Osterrieth Complex Figure (Caffarra et al., 2002) [104]—recall Corsi learning suvra-span (Spinnler e Tognoni, 1987) [105]	Rey–Osterrieth Complex Figure (Caffarra et al., 2002) [104]—recall Corsi learning suvra-span (Spinnler e Tognoni, 1987) [105]	Corsi learning suvra-span (Spinnler e Tognoni, 1987) [105]
**Language** ***Comprehension***	Neuropsychological Examination for Aphasia (ENPA)—words and sentences comprehension test (Capasso and Miceli, 2001) [106]		Token Test (Spinnler e Tognoni, 1987) [105]
***Naming***		Naming colored pictures (Catricalà et al., 2013) [107] Naming figures (Catricalà et al., 2017) [108]	Naming colored pictures (Catricalà et al., 2013) [107]
***Fluency***	Phonemic Fluency Test and Semantic Fluency Test (Costa et al., 2014) [109]	Phonemic Fluency Test and Semantic Fluency Test (Costa et al., 2014) [109]	Phonemic Fluency Test and Semantic Fluency Test (Costa et al., 2014) [109]
**Visual–constructive skills** **Visual–perceptive functions**	Rey–Osterrieth Complex Figure (Caffarra et al., 2002)—copy [104]	Clock Drawing Test (Caffarra et al., 2011) [110] Rey–Osterrieth Complex Figure (Caffarra et al., 2002)—copy [104]	Clock Drawing Test (Caffarra et al., 2011) [110]
**Visual agnosia**			Street’s Completion Test (Spinnler e Tognoni, 1987) [105]
**Constructive apraxia**			Constructive Apraxia Test (Spinnler e Tognoni, 1987) [105]
**Attention** ***Selective***	Visual Search Test (Spinnler e Tognoni, 1987) [105]	Visual Search Test (Della Sala et al., 1992) [111]	Visual Search Test (Spinnler e Tognoni, 1987) [105]
***Attention/executive functions***		Trail Making Test (Siciliano et al., 2019) [112] Stroop Color Word Interference Test (Caffarra et al., 2002) [113]	Trail Making Test (Siciliano et al., 2019) [112]
**Executive functions** ***Global executive efficiency***	Frontal Assessment Battery (Apollonio et al., 2005) [114]		Frontal Assessment Battery (Aiello et al., 2022) [115]
***Verbal Fluency***	Phonemic Fluency Test (Costa et al., 2014) [109]	Phonemic Fluency Test (Costa et al., 2014) [109]	Alternate Fluency Test (Costa et al., 2014) [109] Phonemic Fluency Test (Costa et al., 2014) [109]
***Planning and organization***	Rey–Osterrieth Complex Figure (Caffarra et al., 2002)—copy [104]	Rey–Osterrieth Complex Figure (Caffarra et al., 2002)—copy [104]	
***Logical reasoning***	Colored Progressive Matrices (Basso et al., 1987) [116]	Colored Progressive Matrices (Carlesimo et al., 1996) [117]	Colored Progressive Matrices (Basso et al., 1987) [116]

## References

[1] Bartlett K.A., Camba J.D. (2023). Gender differences in spatial ability: A critical review. Educ. Psychol. Rev..

[2] Wolbers T., Hegarty M. (2010). What determines our navigational abilities?. Trends Cogn. Sci..

[3] Piantadosi S.T. (2021). The computational origin of representation. Minds Mach..

[4] Lester A.W., Moffat S.D., Wiener J.M., Barnes C.A., Wolbers T. (2017). The aging navigational system. Neuron.

[5] Burgess N. (2008). Spatial cognition and the brain. Ann. N. Y. Acad. Sci..

[6] Coughlan G., Laczó J., Hort J., Minihane A.M., Hornberger M. (2018). Spatial navigation deficits-overlooked cognitive marker for preclinical Alzheimer disease?. Nat. Rev. Neurol..

[7] Laczó M., Martinkovic L., Lerch O., Wiener J.M., Kalinova J., Matuskova V., Nedelska Z., Vyhnalek M., Hort J., Laczó J. (2022). Different profiles of spatial navigation deficits in Alzheimer’s disease biomarker-positive versus biomarker-negative older adults with amnestic mild cognitive impairment. Front. Aging Neurosci..

[8] Poulter S., Hartley T., Lever C. (2018). The neurobiology of mammalian navigation. Curr. Biol..

[9] Warren W.H. (2019). Non-Euclidean navigation. J. Exp. Biol..

[10] Adamo D.E., Briceño E.M., Sindone J.A., Alexander N.B., Moffat S.D. (2012). Age differences in virtual environment and real world. path integration. Front. Aging Neurosci..

[11] Chersi F., Burgess N. (2015). The cognitive architecture of spatial navigation: Hippocampal and striatal contributions. Neuron.

[12] Harootonian S.K., Ekstrom A.D., Wilson R.C. (2022). Combination and competition between path integration and landmark navigation in the estimation of heading direction. PLoS Comput. Biol..

[13] Jeffery K.J. (2007). Self-localization and the entorhinal–hippocampal system. Curr. Opin. Neurobiol..

[14] Noel J.P., Angelaki D.E. (2022). Cognitive, systems, and computational neurosciences of the self in motion. Annu. Rev. Psychol..

[15] Ekstrom A.D., Hill P.F. (2023). Spatial navigation and memory: A review of the similarities and differences relevant to brain models and age. Neuron.

[16] Tuena C., Serino S., Stramba-Badiale C., Pedroli E., Goulene K.M., Stramba-Badiale M., Riva G. (2023). Usability of an Embodied CAVE System for Spatial Navigation Training in Mild Cognitive Impairment. J. Clin. Med..

[17] Santoro I., Murgia M., Sors F., Agostini T. (2017). Walking reduces the gap between encoding and sensorimotor alignment effects in spatial updating of described environments. Q. J. Exp. Psychol..

[18] Vantomme G., Rovó Z., Cardis R., Béard E., Katsioudi G., Guadagno A., Perrenoud V., Fernandez L.M., Lüthi A. (2020). A thalamic reticular circuit for head direction cell tuning and spatial navigation. Cell Rep..

[19] Bin Khalid I., Reifenstein E.T., Auer N., Kunz L., Kempter R. (2022). Quantitative modeling of the emergence of macroscopic grid-like representations. bioRxiv.

[20] Lever C., Burton S., Jeewajee A., O’Keefe J., Burgess N. (2009). Boundary vector cells in the subiculum of the hippocampal formation. J. Neurosci..

[21] O’Keefe J., Burgess N., Donnett J.G., Jeffery K.J., Maguire E.A. (1998). Place cells, navigational accuracy, and the human hippocampus. Philos. Trans. R. Soc. Lond. B Biol. Sci..

[22] Dong C., Madar A.D., Sheffield M.E. (2021). Distinct place cell dynamics in CA1 and CA3 encode experience in new environments. Nat. Commun..

[23] Grieves R.M., Jedidi-Ayoub S., Mishchanchuk K., Liu A., Renaudineau S., Jeffery K.J. (2020). The place-cell representation of volumetric space in rats. Nat. Commun..

[24] Danjo T. (2020). Allocentric representations of space in the hippocampus. Neurosci. Res..

[25] Puthusseryppady V. (2021). Spatial Disorientation in Alzheimer’s Disease: The Role of Spatial Navigation Impairments and the Outdoor Environment. Ph.D. Thesis.

[26] Rowe J.B., Stephan K.E., Friston K., Frackowiak R.S., Passingham R.E. (2005). The prefrontal cortex shows context-specific changes in effective connectivity to motor or visual cortex during the selection of action or colour. Cereb. Cortex.

[27] Hampel H., Shaw L.M., Aisen P., Chen C., Lleó A., Iwatsubo T., Iwata A., Yamada M., Ikeuchi T., Jia J. (2022). State-of-the-art of lumbar puncture and its place in the journey of patients with Alzheimer’s disease. Alzheimers Dement..

[28] Edwards M., Corkill R. (2023). Disease-modifying treatments in Alzheimer’s disease. J. Neurol..

[29] Gazova I., Vlcek K., Laczó J., Nedelska Z., Hyncicova E., Mokrisova I., Sheardova K., Hort J. (2012). Spatial navigation—A unique window into physiological and pathological aging. Front. Aging Neurosci..

[30] Gazova I., Laczó J., Rubinova E., Mokrisova I., Hyncicova E., Andel R., Vyhnalek M., Sheardova K., Coulson E.J., Hort J. (2013). Spatial navigation in young versus older adults. Front. Aging Neurosci..

[31] Zhong J.Y., Moffat S.D. (2016). Age-related differences in associative learning of landmarks and heading directions in a virtual navigation task. Front. Aging Neurosci..

[32] Benke T., Karner E., Petermichl S., Prantner V., Kemmler G. (2014). Neuropsychological deficits associated with route learning in Alzheimer disease, MCI, and normal aging. Alzheimer Dis. Assoc. Disord..

[33] Borghesani P.R., Johnson L.C., Shelton A.L., Peskind E.R., Aylward E.H., Schellenberg G.D., Cherrier M.M. (2008). Altered medial temporal lobe responses during visuospatial encoding in healthy APOE*4 carriers. Neurobiol. Aging.

[34] Slot R.E., Sikkes S.A., Berkhof J., Brodaty H., Buckley R., Cavedo E., Dardiotis E., Guillo-Benarous F., Hampel H., Kochan N.A. (2019). Subjective cognitive decline and rates of incident Alzheimer’s disease and non–Alzheimer’s disease dementia. Alzheimers Dement..

[35] Cerman J., Ross A., Laczo J., Martin V., Zuzana N., Ivana M., Katerina S., Jakub H. (2018). Subjective Spatial navigation complaints-a frequent symptom reported by patients with subjective cognitive decline, mild cognitive impairment and Alzheimer’s disease. Curr. Alzheimer Res..

[36] Tuena C., Mancuso V., Stramba-Badiale C., Pedroli E., Stramba-Badiale M., Riva G., Repetto C. (2021). Egocentric and allocentric spatial memory in mild cognitive impairment with real-world and virtual navigation tasks: A systematic review. JAD.

[37] Rabin L.A., Smart C.M., Amariglio R.E. (2017). Subjective cognitive decline in preclinical Alzheimer’s disease. Annu. Rev. Clin. Psychol..

[38] Buchhave P., Minthon L., Zetterberg H., Wallin Å.K., Blennow K., Hansson O. (2012). Cerebrospinal fluid levels ofβ-amyloid 1-42, but not of tau, are fully changed already 5 to 10 years before the onset of Alzheimer dementia. Arch. Gen. Psychiatry.

[39] Ferreira D., Verhagen C., Hernández-Cabrera J.A., Cavallin L., Guo C.J., Ekman U., Muehlboeck J.-S., Simmons A., Barroso J., Wahlund L.-O. (2017). Distinct subtypes of Alzheimer’s disease based on patterns of brain atrophy: Longitudinal trajectories and clinical applications. Sci. Rep..

[40] Braak H., del Tredici K. (2015). The preclinical phase of the pathological process underlying sporadic Alzheimer’s disease. Brain.

[41] Liesinger A.M., Graff-Radford N.R., Duara R., Carter R.E., Hanna Al-Shaikh F.S., Koga S., Hinkle K.M., DiLello S.K., Johnson M.F., Aziz A. (2018). Sex and age interact to determine clinicopathologic differences in Alzheimer’s disease. Acta Neuropathol..

[42] Markesbery W.R. (2010). Neuropathologic alterations in mild cognitive impairment: A review. JAD.

[43] Bierbrauer A., Kunz L., Gomes C.A., Luhmann M., Deuker L., Getzmann S., Wascher E., Gajewski P.D., Hengstler J.G., Fernandez-Alvarez M. (2020). Unmasking selective path integration deficits in Alzheimer’s disease risk carriers. Sci. Adv..

[44] Braak H., del Tredici K. (2011). The pathological process underlying Alzheimer’s disease in individuals under thirty. Acta Neuropathol..

[45] Mattsson N., Groot C., Jansen W.J., Landau S.M., Villemagne V.L., Engelborghs S., Mintun M.M., Lleo A., Molinuevo J.L., Jagust W.J. (2018). Prevalence of the apolipoprotein E ε4 allele in amyloid β positive subjects across the spectrum of Alzheimer’s disease. Alzheimers Dement..

[46] Plaza-Rosales I., Brunetti E., Montefusco-Siegmund R., Madariaga S., Hafelin R., Ponce D.P., Behrens M.I., Maldonado P.L., Paula-Lima A. (2023). Visual-spatial processing impairment in the occipital-frontal connectivity network at early stages of Alzheimer’s disease. Front. Aging Neurosci..

[47] Brewer A.A., Barton B. (2014). Visual cortex in aging and Alzheimer’s disease: Changes in visual field maps and population receptive fields. Front. Psychol..

[48] Godfrey M.E., Wojcik D.P., Krone C.A. (2003). Apolipoprotein E genotyping as a potential biomarker for mercury neurotoxicity. JAD.

[49] Liu C.C., Kanekiyo T., Xu H., Bu G. (2013). Apolipoprotein E and Alzheimer disease: Risk mechanisms and therapy. Nat. Rev. Neurol..

[50] Eisenberg D.T., Kuzawa C.W., Hayes M.G. (2010). Worldwide allele frequencies of the human apolipoprotein E gene: Climate, local adaptations, and evolutionary history. Am. J. Phys. Anthropol..

[51] Yamazaki Y., Zhao N., Caulfield T.R., Liu C.C., Bu G. (2019). Apolipoprotein E and Alzheimer disease: Pathobiology and targeting strategies. Nat. Rev. Neurol..

[52] Carmona S., Hardy J., Guerreiro R. (2018). The genetic landscape of Alzheimer disease. Handb. Clin. Neurol..

[53] Corder E.H., Saunders A.M., Risch N.J., Strittmatter W.J., Schmechel D.E., Gaskell P.C., Rimmler J.B., Locke P.A., Conneally P.M., Schmader K.E. (1994). Protective effect of apolipoprotein E type 2 allele for late onset Alzheimer disease. Nat. Genet..

[54] Di Battista M.A., Heinsinger N., William Rebeck G. (2016). Alzheimer’s disease genetic risk factor APOE-ε4 also affects normal brain function. Curr. Alzheimer Res..

[55] Reiman E.M., Caselli R.J., Yun L.S., Chen K., Bandy D., Minoshima S., Thibodeau S.N., Osborne D. (1996). Preclinical evidence of Alzheimer’s disease in persons homozygous for the ε4 allele for apolipoprotein E. NEJM.

[56] Mendonça A.R., Loureiro L.M., Nórte C.E., Landeira-Fernandez J. (2022). Episodic memory training in elderly: A systematic review. Front. Psychol..

[57] Serino S., Morganti F., di Stefano F., Riva G. (2015). Detecting early egocentric and allocentric impairments deficits in Alzheimer’s disease: An experimental study with virtual reality. Front. Aging Neurosci..

[58] Zanco M., Plácido J., Marinho V., Ferreira J.V., de Oliveira F., Monteiro-Junior R., Barca M., Engedal K., Laks J., Deslandes A. (2018). Spatial navigation in the elderly with Alzheimer’s disease: A cross-sectional study. J. Alzheimers Dis..

[59] Tu S., Wong S., Hodges J.R., Irish M., Piguet O., Hornberger M. (2015). Lost in spatial translation–A novel tool to objectively assess spatial disorientation in Alzheimer’s disease and frontotemporal dementia. Cortex.

[60] Yew B., Alladi S., Shailaja M., Hodges J.R., Hornberger M. (2013). Lost and forgotten? Orientation versus memory in Alzheimer’s disease and frontotemporal dementia. J. Alzheimers Dis..

[61] Claessen M.H.G., van der Ham I.J.M. (2017). Classification of navigation impairment: A systematic review of neuropsychological case studies. Neurosci. Biobehav. Rev..

[62] Fu H., Rodriguez G.A., Herman M., Emrani S., Nahmani E., Barrett G., Figueroa H.Y., Goldberg E., Hussaini S.A., Duff K.E. (2017). Tau pathology induces excitatory neuron loss, grid cell dysfunction, and spatial memory deficits reminiscent of early Alzheimer’s disease. Neuron.

[63] Prince S.M., Paulson A.L., Jeong N., Zhang L., Amigues S., Singer A.C. (2021). Alzheimer’s pathology causes impaired inhibitory connections and reactivation of spatial codes during spatial navigation. Cell Rep..

[64] Puthusseryppady V., Morrissey S., Spiers H., Patel M., Hornberger M. (2022). Predicting real world spatial disorientation in Alzheimer’s disease patients using virtual reality navigation tests. Sci. Rep..

[65] MacAndrew M., Brooks D., Beattie E. (2019). NonPharmacological interventions for managing wandering in the community: A narrative review of the evidence base. Health Soc. Care Community.

[66] Bowen M.E., McKenzie B., Steis M., Rowe M. (2011). Prevalence of and antecedents to dementia-related missing incidents in the community. Dement. Geriatr. Cogn. Disord..

[67] Lau W.M., Chan T.Y., Szeto S.L. (2019). Effectiveness of a home-based missing incident prevention program for community-dwelling elderly patients with dementia. Int. Psychogeriatr..

[68] Sheehan B., Burton E., Mitchell L. (2006). Outdoor wayfinding in dementia. Dementia.

[69] Muffato V., Borella E., Pazzaglia F., Meneghetti C. (2022). Orientation Experiences and Navigation Aid Use: A Self-Report Lifespan Study on the Role of Age and Visuospatial Factors. Int. J. Environ. Res. Public Health.

[70] Lithfous S., Dufour A., Després O. (2013). Spatial navigation in normal aging and the prodromal stage of Alzheimer’s disease: Insights from imaging and behavioral studies. Ageing Res. Rev..

[71] Nedelska Z., Andel R., Laczó J., Vlcek K., Horinek D., Lisy J., Sheardova K., Bureš J., Hort J. (2012). Spatial navigation impairment is proportional to right hippocampal volume. Proc. Natl. Acad. Sci. USA.

[72] Laczó J., Andel R., Vlček K., Macoška V., Vyhnálek M., Tolar M., Bojar M., Hort J. (2011). Spatial navigation and APOE in amnestic mild cognitive impairment. Neurodegener. Dis..

[73] Howett D., Castegnaro A., Krzywicka K., Hagman J., Marchment D., Henson R., Rio M., King J.A., Burgess N., Chan D. (2019). Differentiation of mild cognitive impairment using an entorhinal cortex-based test of virtual reality navigation. Brain.

[74] Serino S., Pedroli E., Tuena C., de Leo G., Stramba-Badiale M., Goulene K., Mariotti N.G., Riva G. (2017). A novel virtual reality-based training protocol for the enhancement of the “mental frame syncing” in individuals with Alzheimer’s disease: A development-of-concept trial. Front. Aging Neurosci..

[75] Cammisuli D.M., Cipriani G., Castelnuovo G. (2022). Technological solutions for diagnosis, management and treatment of Alzheimer’s disease-related symptoms: A structured review of the recent scientific literature. Int. J. Environ. Res. Public Health.

[76] Thaler R.H., Sunstein C.R. (2009). Nudge: Improving Decisions about Health, Wealth and Happiness.

[77] Cammisuli D.M., Pietrabissa G., Castelnuovo G. (2021). Improving wellbeing of community-dwelling people with mild cognitive impairment: The SENIOR [SystEm of Nudge theory based ICT applications for OldeR citizens] project. Neural Regen. Res..

[78] Ghosh A., Puthusseryppady V., Chan D., Mascolo C., Hornberger M. (2022). Machine learning detects altered spatial navigation features in outdoor behaviour of Alzheimer’s disease patients. Sci. Rep..

[79] Jessen F., Amariglio R.E., Buckley R.F., van der Flier W.M., Han Y., Molinuevo J.L., Rabin L., Rentz D.M., Rodriguez-Gomez O., Saykin A.J. (2020). The characterisation of subjective cognitive decline. Lancet Neurol..

[80] Dubois B., Feldman H.H., Jacova C., Hampel H., Molinuevo J.L., Blennow K., DeKosky S.T., Gauthier S., Selkoe D., Bateman R. (2014). Advancing research diagnostic criteria for Alzheimer’s disease: The IWG-2 criteria. Lancet Neurol..

[81] Solfrizzi V., Panza F., Colacicco A.M., D’Introno A., Capurso C., Torres F., Grigoletto F., Maggi S., del Parigi A., Reiman E.M. (2004). Vascular risk factors, incidence of MCI, and rates of progression to dementia. Neurology.

[82] Mauri M., Sinforiani E., Zucchella C., Cuzzoni M.G., Bono G. (2012). Progression to dementia in a population with amnestic mild cognitive impairment: Clinical variables associated with conversion. Funct. Neurol..

[83] Moretti F., de Ronchi D., Palmer K., Forlani C., Morini V., Ferrari B., Dalmonte E., Atti A.R. (2013). Prevalence and characteristics of mild cognitive impairment in the general 832 population. Data from an Italian population-based study: The Faenza Project. Aging Ment. Health.

[84] Mondini S., Pucci V., Montemurro S., Rumiati R.I. (2022). Protective factors for subjective cognitive decline individuals: Trajectories and changes in a longitudinal study with Italian elderly. Eur. J. Neurol..

[85] Chiari L., van Lummel R., Becker C., Pfeiffer K., Lindemann U., Zijlstra W. (2009). Deliverable 2.2: Classification of the User’s Needs, Characteristics and Scenarios—Update. [Rapporto Non Pubblicato Del Progetto UE [6° Programma Quadro, Contratto IST n. 045622] Sensing and Action to Support Mobility in Ambient Assisted Living].

[86] Champely S., Ekstrom C., Dalgaard P., Gill J., Weibelzahl S., Anandkumar A., Ford C., Volcic R., de Rosario H., de Rosario M.H. (2020). Package ‘Pwr’.

[87] Rothstein H.R., Borenstein M., Cohen J., Pollack S. (1990). Statistical power analysis for multiple regression/correlation: A computer program. Educ. Psychol. Meas..

[88] Schaat S., Koldrack P., Yordanova K., Kirste T., Teipel S. (2020). Real-time detection of spatial disorientation in persons with mild cognitive impairment and dementia. Gerontology.

[89] Levine T.F., Allison S.L., Stojanovic M., Fagan A.M., Morris J.C., Head D. (2020). Spatial navigation ability predicts progression of dementia symptomatology. Alzheimers Dement..

[90] Schöberl F., Pradhan C., Irving S., Buerger K., Xiong G., Kugler G., Kohlbecher S., Engmann D.P.J., Werner P., Brendel M. (2020). Real-space navigation testing differentiates between amyloid-positive and-negative aMCI. Neurology.

[91] Colmant L., Bierbrauer A., Bellaali Y., Kunz L., van Dongen J., Sleegers K., Axmacher N., Lefèvre P., Hanseeuw B. (2023). Dissociating effects of aging and genetic risk of sporadic Alzheimer’s disease on path integration. Neurobiol. Aging.

[92] Sheardova K., Laczó J., Vyhnalek M., Mokrisova I., Telensky P., Andel R. (2015). Spatial navigation complaints are associated with anxiety regardless of the real performance in non-demented elderly. J. Depress. Anxiety.

[93] Pawlaczyk N., Szmytke M., Meina M., Lewandowska M., Stępniak J., Bałaj B., Dreszer J. (2021). Gait Analysis under Spatial Navigation Task in Elderly People—A Pilot Study. Sensors.

[94] Plácido J., de Almeida C.A.B., Ferreira J.V., de Oliveira Silva F., Monteiro-Junior R.S., Tangen G.G., Laks J., Deslandes A.C. (2022). Spatial navigation in older adults with mild cognitive impairment and dementia: A systematic review and meta-analysis. Exp. Gerontol..

[95] Crean S., Ward A., Mercaldi C.J., Collins J.M., Cook M.N., Baker N.L., Arrighi H.M. (2011). Apolipoprotein E ε4 prevalence in Alzheimer’s disease patients varies across global populations: A systematic literature review and meta-analysis. Dement. Geriatr. Cogn. Disord..

[96] Wang L., Jiao Y., Zhao A., Xu X., Ye G., Zhang Y., Wang Y., Deng Y., Xu W., Liu J. (2022). Analysis of Genetic Association Between ABCA7 Polymorphism and Alzheimer’s Disease Risk in the Southern Chinese Population. Front. Aging Neurosci..

[97] Raulin A.C., Doss S.V., Trottier Z.A., Ikezu T.C., Bu G., Liu C.C. (2022). ApoE in Alzheimer’s disease: Pathophysiology and therapeutic strategies. Mol. Neurodegener..

[98] Magni E., Binetti G., Bianchetti A., Rozzini R., Trabucchi M. (1996). Mini-Mental State Examination: A normative study in Italian elderly population. Eur. J. Neurol..

[99] Foderaro G., Isella V., Mazzone A., Biglia E., di Gangi M., Pasotti F., Sansotera F., Grobberio M., Raimondi V., Mapelli C. (2022). Brand new norms for a good old test: Northern Italy normative study of MiniMental State Examination. Neurol. Sci..

[100] Aiello E.N., Gramegna C., Esposito A., Gazzaniga V., Zago S., Difonzo T., Maddaluno O., Appollonio I., Bolognini N. (2022). The Montreal Cognitive Assessment (MoCA): Updated norms and psychometric insights into adaptive testing from healthy individuals in Northern Italy. Aging Clin. Exp. Res..

[101] Monaco M., Costa A., Caltagirone C., Carlesimo G.A. (2013). Forward and backward span for verbal and visuo-spatial data: Standardization and normative data from an Italian adult population. Neurol. Sci..

[102] Mauri M., Carlesino G.A., Graceffa A.M., Loasses A., Lorusso S., Sinforiani E., Bono G., Caltagirone C. (1997). Standardizzazione di due nuovi test di memoria: Apprendimento di liste di parole correlate e non correlate semanticamente. Arch. Psicol. Neurol. Psichiatr..

[103] Girtler N., de Carli F., Amore M., Arnaldi D., Bosia L.E., Bruzzaniti C., Cappa S.F., Cocito L., Colazzo G., Ghio L. (2015). A normative study of the Italian printed word version of the free and cued selective reminding test. Neurol. Sci..

[104] Caffarra P., Vezzadini G., Dieci F., Zonato F., Venneri A. (2002). Rey-Osterrieth complex figure: Normative values in an Italian population sample. Neurol. Sci..

[105] Spinnler H., Tognoni G. (1987). Italian Group on the Neuropsychological Study of Ageing: Italian standardization and classification of neuropsychological tests. Ital. J. Neurol. Sci..

[106] Capasso R., Miceli G. (2001). Esame Neuropsicologico per l’Afasia: ENPA.

[107] Catricalà E., della Rosa P.A., Ginex V., Mussetti Z., Plebani V., Cappa S.F. (2013). An Italian battery for the assessment of semantic memory disorders. Neurol. Sci..

[108] Catricalà E., Gobbi E., Battista P., Miozzo A., Polito C., Boschi V., Esposito V., Cuoco S., Barone P., Sorbi S. (2017). SAND: A Screening for Aphasia in NeuroDegeneration. Development and normative data. Neurol. Sci..

[109] Costa A., Bagoj E., Monaco M., Zabberoni S., de Rosa S., Papantonio A.M., Mundi C., Caltagirone C., Carlesimo G.A. (2014). Standardization and normative data obtained in the Italian population for a new verbal fluency instrument, the phonemic/semantic alternate fluency test. Neurol. Sci..

[110] Caffarra P., Gardini S., Zonato F., Concari L., Dieci F., Copelli S., Freedman M., Stracciari A., Venneri A. (2011). Italian norms for the Freedman version of the Clock Drawing Test. J. Clin. Exp. Neuropsychol..

[111] Della Sala S., Laiacona M., Spinnler H., Ubezio C. (1992). A cancellation test: Its reliability in assessing attentional deficits in Alzheimer’s disease. Psychol. Med..

[112] Siciliano M., Chiorri C., Battini V., Sant’Elia V., Altieri M., Trojano L., Santangelo G. (2019). Regression-based normative data and equivalent scores for Trail Making Test (TMT): An updated Italian normative study. Neurol. Sci..

[113] Caffarra P., Vezzadini G., Dieci F., Zonato F., Venneri A. (2002). A short version of the Stroop test: Normative data in an Italian population sample. Riv. Neurol..

[114] Appollonio I., Leone M., Isella V., Piamarta F., Consoli T., Villa M.L., Forapani E., Russo A., Nichelli P. (2005). The Frontal Assessment Battery (FAB): Normative values in an Italian population sample. Neurol. Sci..

[115] Aiello E.N., Esposito A., Gramegna C., Gazzaniga V., Zago S., Difonzo T., Appollonio I.M., Bolognini N. (2022). The Frontal Assessment Battery (FAB) and its sub-scales: Validation and updated normative data in an Italian population sample. Neurol. Sci..

[116] Basso A., Capitani E., Laiacona M. (1987). Raven’s coloured progressive matrices: Normative values on 305 adult normal controls. Funct. Neurol..

[117] Carlesimo G.A., Caltagirone C., Gainotti G.U., Fadda L., Gallassi R., Lorusso S., Marfia G., Marra C.A., Nocentini U., Panetti L. (1996). The mental deterioration battery: Normative data, diagnostic reliability and qualitative analyses of cognitive impairment. Eur. Neurol..

